# Minimized Soft Tissue Release in Robotic-Assisted Total Knee Arthroplasty: A Retrospective Review of 100 Cases

**DOI:** 10.7759/cureus.68062

**Published:** 2024-08-28

**Authors:** Sanjay B Londhe, Supreet Bajwa, Ravi Teja Rudraraju, Ravi Shah, Kunal Patel, Suneet Velankar, Govindkumar Baranwal, Zara Namjoshi

**Affiliations:** 1 Department of Orthopaedics, Criticare Asia Hospital, Mumbai, IND; 2 Department of Orthopaedics, Wockhardt Hospital, Mumbai, IND; 3 Department of Orthopaedics, Apollo Hospitals, Hyderabad, IND; 4 Department of Statistics, Criticare Asia Hospital, Mumbai, IND

**Keywords:** valgus deformity, varus deformity, gap balancing, pre-operative planning, soft tissue release, robotic knee surgery, total knee arthroplasty technique

## Abstract

Aim

For a successful total knee arthroplasty (TKA), bone cuts and soft tissue envelope must be balanced to ensure equal flexion and extension gaps. The study aims to assess if preoperative computed tomography (CT) scans and planning software reduce soft tissue release.

Methodology

A retrospective analysis was conducted for the first 100 consecutive robotic-assisted (RA) TKA (RA-TKA) patients between March 2022 and May 2023. All patients underwent preoperative leg CT scans utilizing a fully automated Cuvis Joint robot. Planning software determined implant sizes and bone resections to achieve a 180° hip-knee-ankle axis. A posterior-stabilized knee design was implanted during surgery by the same surgical team using a medial parapatellar approach. The study hypothesis was, that RA-TKA with preoperative CT scans and planning does not reduce soft tissue release incidence, comparing it with the historical control cohort using chi-square tests (*P *< 0.05 considered significant).

Results

The study consisted of 89 women and 11 males, with an average age of 65.3 ± 12 years. The average body mass index of the patients was 27.4 ± 5.2 kg/m^2^. Ninety-four individuals had varus knees, while six had valgus knee deformity. Varus deformity ranged between 7° and 18°, and valgus knee deformity ranged from 6° to 14° preoperatively. Twelve patients (12.77%) of 94 varus knees (versus historic control 29%, *P*-value = 0.0047) and one out of 6 (16.67%) valgus knees (versus historic control 84%, *P *< 0.0001) required posteromedial and posterolateral release for appropriate knee balance.

Conclusions

The study negates the null hypothesis and indicates that RA-TKA with preoperative CT scans and planning reduces the incidence of soft tissue releases to achieve a well-balanced knee.

## Introduction

Total knee arthroplasty (TKA) stands as a cornerstone in the management of debilitating knee osteoarthritis (OA), providing relief to countless patients worldwide [1]. The success of TKA, however, is intricately tied to the attainment of a well-balanced knee, a key determinant of postoperative function, implant stability, and patient satisfaction [1]. Achieving optimal soft tissue balance is a critical aspect of TKA, as improper alignment can lead to complications such as instability, premature wear, and reduced implant longevity [1].

The potential for compromised knee joint stability and heightened risk of problems may arise from an excessive release of soft tissue [2,3]. Historically, during TKA, the attainment of soft tissue balance has frequently required the intervention of ligament, and soft tissue release is a matter of individual surgeon discretion and is often seen as a subjective process. Consequently, it may not serve as a reliable indicator for assessing soft tissue conditions in TKA [3]. The pursuit of achieving a delicate equilibrium between attaining appropriate alignment and reducing the need for soft tissue release has spurred advancements in surgical methodologies and technologies, such as robotic-assisted (RA)-TKA [4]. Robotic systems, which are equipped with sophisticated planning software and preoperative imaging, provide surgeons with an unprecedented level of precision and accuracy [4]. The use of preoperative computed tomography (CT) scans and planning software in the workflow of RA-TKA is intended to enhance the optimization of implant alignment, size, and location [4]. This prompts an inquiry into whether these innovations have the potential to reduce the necessity for soft tissue release, therefore augmenting the overall efficacy of TKA.

This study builds upon the evolving landscape of RA-TKA by retrospectively examining the outcomes of 100 patients who underwent TKA with a posterior-stabilized (PS) knee design using an automated robotic system. The investigation focuses on the reduction of soft tissue release by leveraging preoperative CT scans and planning software. By shedding light on the potential benefits of this approach, we aim to contribute to the growing body of evidence guiding orthopedic surgeons toward more refined and patient-specific TKA procedures.

The part of data from the study was presented as a *poster presentation* during the World Arthroplasty Congress (organized by the European Knee and Hip Society, Knee, and Hip Society, United States) in Madrid, Spain from April 18 to 19, 2024.

## Materials and methods

A retrospective analysis of the first 100 patients was conducted at a tertiary center in India between March 2022 and May 2023. Patients undergoing RA primary TKA for advanced arthritis were included in the study. Preoperative knee deformities were categorized, with patients presenting varus or valgus alignment. Patients who previously had knee surgery were excluded from the study. This study adhered to ethical guidelines, and all patients provided written informed consent before the surgery. Due to the retrospective nature of the study, the local Research ethics committee waived the approval.

Preoperative planning and imaging

All patients underwent preoperative CT scans to meticulously evaluate knee alignment and assess the extent of deformities. The data obtained from the CT scans were then imported into advanced planning software (J-Planner), providing a three-dimensional (3D) visualization of the knee anatomy. The planning software aided in determining the optimal levels of bone resection and sizing of TKA components, necessary to achieve a hip-knee-axis (HKA) of 180°.

RA-TKA

The implementation of a fully automated robotic system in surgical procedures enabled the accurate implementation of preoperative plans. The Cuvis joint robot facilitated intraoperative modifications guided by real-time feedback, thereby augmenting the precision of implant positioning and alignment [5].

**Figure 1:**
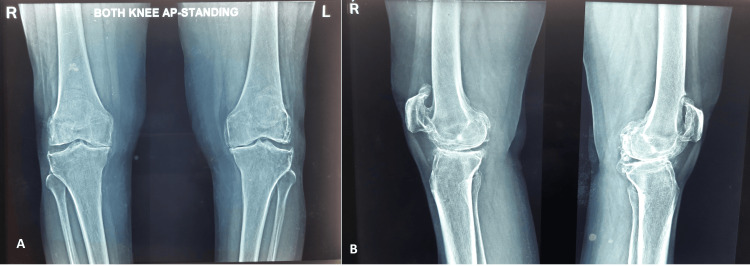
(A and B) Preoperative radiographs of the diseased knee.

**Figure 2:**
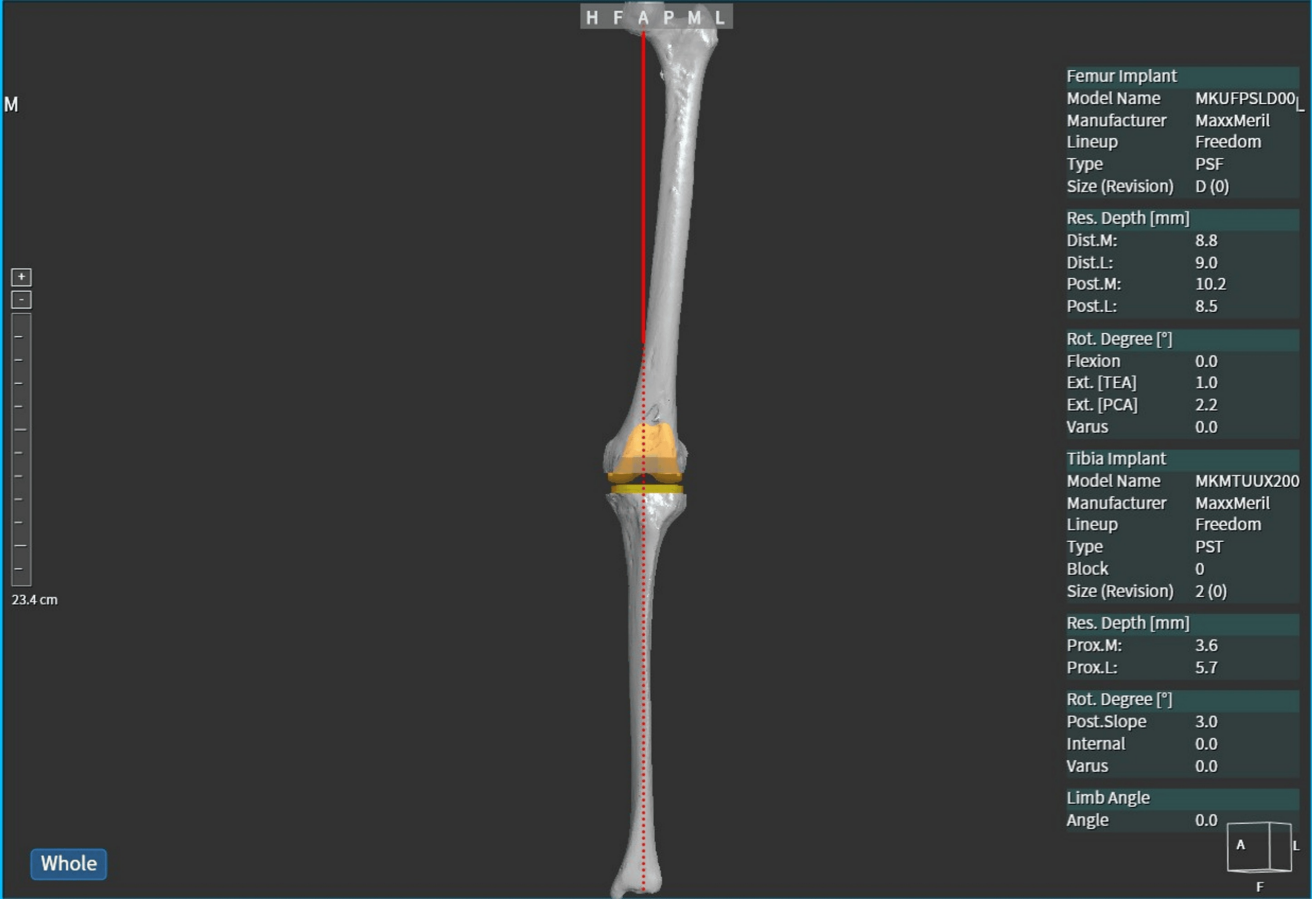
Preoperative planning performed using J-Planner of fully automated Cuvis Joint robot system.

The software facilitates the generation of a 3D representation of the knee joint through an automated process of segmenting the CT images. Based on the 3D scans, the surgeon engages in preoperative planning and establishes subsequent parameters, such as the point of rotation for the HKA joints, the existence of skeletal malformation, the dimensions, and placement of implants, the number of osteotomies necessary as determined by the mechanical axis in both the frontal and sagittal planes, the process of identifying and delineating the rotation of the femur and tibia within the axial plane, and the number of registration points necessary for intraoperative surface registration on the femur and tibia [6]. The program processes the inputs provided by the surgeon and generates a comprehensive alignment report utilizing numerical data (Figures 1A-1B; Figure 2).

**Figure 3:**
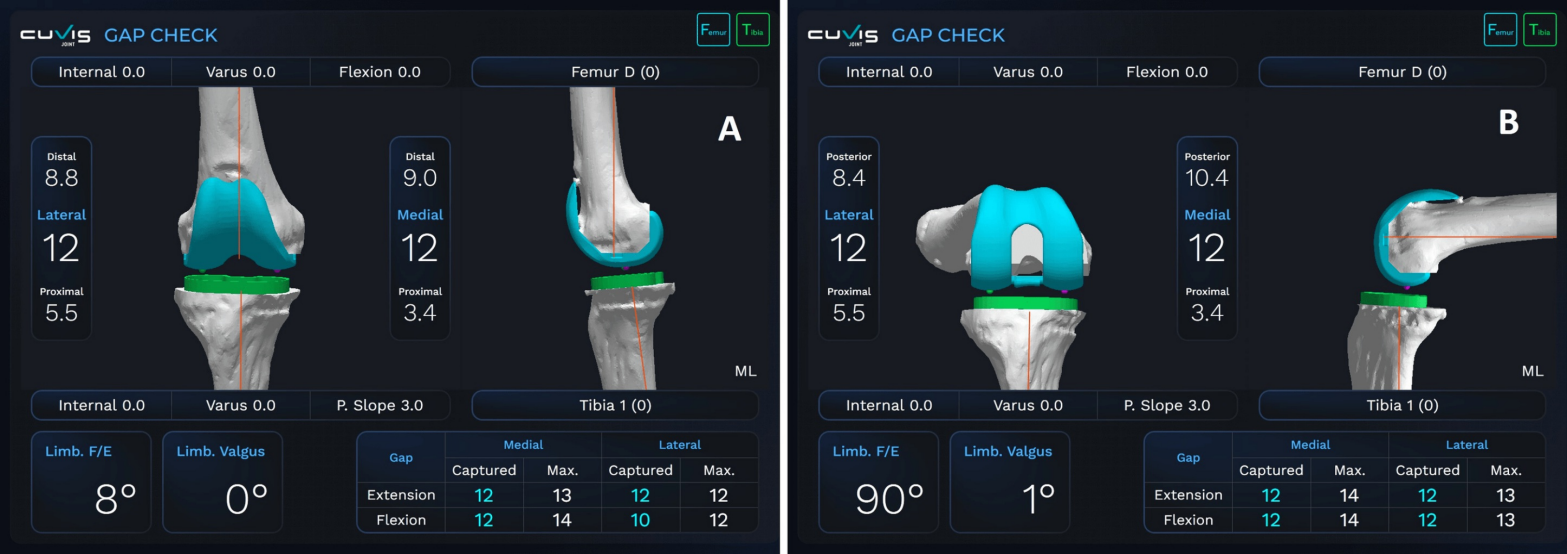
Intraoperative gap balance check in full extension (A) and 90° of flexion (B).

Subsequently, this report was uploaded to the console of the robotic arm. During the surgical procedure, the technician undertook the calibration process of the robot to optimize its ability to manipulate and operate the arm within a predetermined 3D operational area. Ultimately, the arm was enveloped in a sterile drape. Following the initial standard exposure, reflective arrays were strategically positioned in the femoral and tibial diaphysis, at a distance of approximately 10 cm from the joint line. This was accomplished by employing a dual-pin system on each side, the diameter of the pins measuring 4 mm, and they were recommended by the manufacturer to be bicortical to minimize the risk of pin loosening. Subsequently, the femur and tibia were subjected to surface registration utilizing a probe. Following this, the computer algorithm produced a simulated 3D representation of the knee, which was then compared and aligned with the CT scans. The precision of the registration process was determined by achieving a final root mean square (RMS) error that was below the threshold of 1. During this period, it was possible to perform minimal soft tissue release, remove osteophytes, and excise the anterior horns of the menisci. The technology provided instantaneous measurements of the gaps across the whole range of motion (ROM), and these gaps were adjusted to achieve a balance that aligns with the surgeon's preferences (Figures 3A-3B).

The knee was immobilized to restrict leg movement and facilitate the robot's ability to monitor the intricate leg movements during the surgery. If the displacement of the movement was above a specific threshold, the cutting arm became immobile. The fully automated Cuvis Joint robot (Curexo, South Korea, supported by Meril Healthcare Pvt. Ltd., India) was equipped with a predefined movement capability of 1 mm.

The resection was conducted using a 6.2 mm burr, accompanied by continuous automated saline irrigation. This method precisely milled the bone at a predetermined depth, guided by the surgeon's input, ensuring accurate bone preparation. The robotic arm, functioning as an automated system, adhered to a predetermined trajectory to perform milling operations on each surface. We utilized PS implants (Freedom Total Knee System, Maxx Orthopaedics Inc., Plymouth Meeting, PA) at our center for all patients. After the completion of all bone resection, the robotic arm was separated from the patient's body through manual intervention. Subsequently, routine operations were carried out, including the removal of any remaining bone islands, removal of posterior osteophytes and menisci, and soft tissue release, if deemed necessary. The assessment of ligament balance was conducted by doing trials based on the results presented on the monitor, after which the definitive implantation of the knee was commenced [7]. 

In each instance, both the femoral and tibial bone resections were oriented at a right angle to the mechanical axis, with no deviation in varus or valgus alignment (0°). The objective of balancing the equation was to achieve a minimum of 19 mm of joint space in either the medial compartment for a valgus deformity or the lateral compartment for a varus deformity. The necessary soft tissue release was performed to achieve balance after bone resection and removal of remaining osteophytes, whenever a tighter joint space was seen, resulting in the acceptance of the opposite compartment.

After the completion and verification of the proximal tibial and distal femoral cuts, the remaining osteophytes were removed, and the extension space was evaluated. The spacer block indicated a space of 19 mm. The objective was to provide enough amount of room within a single compartment to accommodate the smallest spacer block while also ensuring that the extension fell within the range of 0° to 3°. In the event of tightness in the medial compartment, a posteromedial release procedure was conducted as necessary to achieve equilibrium and adequately accommodate the spacer in both compartments. In cases when the lateral compartment exhibits tightness, a fenestrated posterolateral capsular release procedure was performed as necessary to attain equilibrium and effectively fit the spacer. The aforementioned releases were duly recorded by an independent observer who was not part of the surgical team.

Soft-tissue release assessment

Soft tissue releases were assessed intraoperatively by an independent investigator using predefined criteria. These criteria included the extent of ligament balance achieved and the necessity for additional releases, ensuring a standardized and reproducible method for evaluation.

In varus knee deformities, posteromedial soft tissue release was considered, while in valgus knee deformities, posterolateral release was performed. The decision to perform soft tissue release was based on achieving a well-balanced knee as determined by the robotic system and confirmed intraoperatively [8,9].

Statistical analysis

Statistical analysis was performed using chi-square tests for categorical variables and Student’s t-tests for continuous variables. A *P*-value of less than 0.05 was considered statistically significant. Comparative analysis between the robotic-assisted TKA group and the historical control group was conducted to evaluate the differences in soft tissue release. The rates of soft-tissue release in varus and valgus deformities were expressed as percentages.

## Results

The retrospective analysis of 100 consecutive patients undergoing RATKA with a PS knee design using a fully automated robotic system revealed notable outcomes, particularly in terms of soft tissue release requirements.

Demographic characteristics

Of the 100 patients, 89 (89%) were females and 11 (11%) were males. Table 1 provides the baseline details of the enrolled patients.

**Table 1:** Baseline and demographic characteristics.

Procedural characteristics	
Total number of subjects (*N*)	100
Male, *N* (%)	11 (11%)
Female, *N* (%)	89 (89%)
Age (Years), mean ± SD	65.3 ± 12.6
Body mass index, mean ± SD (kg/m^2^)	27.4 ± 5.2

Preoperative knee deformities were categorized, with 94 patients (94%) presenting varus alignment (range from 7° to 18°) and 6 patients (6%) presenting valgus alignment (range from 6° to 14°) (Table 2). Details of the various sizes of implants used are presented in Table 2.

**Table 2:** Details of the preoperative deformity and size of the posterior stabilized knee implant utilized.

Procedural characteristics		
Total no. of subjects (*N*)	100
Varus knees, *N* (range)	94 (7°-18°)
Valgus knees, *N* (range)	6 (6°-14°)
Left knees implanted, *N* (%)	48 (48%)
Right knees implanted, *N* (%)	52 (52%)
Implant-femoral size (posterior stabilized), *N* (%)	
B	30 (30%)
C	54 (54%)
D	9 (9%)
E	4 (4%)
F	3 (3%)
Implant-tibia size, *N* (%)	
1	34 (34%)
2	39 (39%)
3	21 (21%)
5	3 (3%)
6	3 (3%)

Preoperative planning and imaging

All patients underwent preoperative CT scans and planning software, facilitating precise evaluation of knee alignment and determination of the optimal levels of bone resection and component sizing (Figures 1A-1B; Figure 2). This approach aimed at achieving a HKA of 180° (accepted intraoperative range was 177°-183° of alignment). The average time taken for preoperative planning, registration, robot docking, bone resection, and the total operative time is given in Table 3.

**Table 3:** Procedural details.

Duration of surgery, mean ± SD	
Time taken for preoperative planning (minutes), mean ± SD	7.6 ± 0.69
Time taken for registration (minutes), mean ± SD	6.98 ± 1.37
Time taken for robot docking plus bone resection (minutes), mean ± SD	18.5 ± 3.47
Total operative time (minutes), mean ± SD	114.13 ± 5.49

Soft-tissue release

One of the 6 patients with valgus knee deformity (16.67%) required a posterolateral soft tissue release to achieve the desired balance. In the varus knee deformity group, consisting of 94 patients, 12 (12.77%) required a posteromedial soft tissue release to achieve a well-balanced knee. Our subset analysis of varus knees yielded a need to perform a posteromedial release in 8 out of 12 patients with a preoperative varus deformity of more than 13°. Table 4 shows the various valgus and varus knee corrections post-robotic TKA where majority of valgus/varus both at extension and flexion was well within 2°.

**Table 4:** Postoperative extension and flexion achieved after robotic-assisted total knee arthroplasty.

Extension	
M. gap	10.19 ± 0.93
L. gap	10.63 ± 1.29
V/V	
Limb. neutral 0°	34 (34%)
Limb. valgus 1°	9 (9%)
Limb. valgus 2°	5 (5%)
Limb. valgus 3°	1 (1%)
Limb. valgus 4°	1 (1%)
Limb. varus 1°	28 (28%)
Limb. varus 2°	20 (20%)
Limb. varus 3°	1 (1%)
Limb. varus 4°	1 (1%)
Flexion	
M. gap	8.94 ± 1.71
L. gap	10.12 ± 1.47
Degree	93.06 ± 7.37
V/V	
0°	31 (31%)
Limb. valgus 1°	12 (12%)
Limb. valgus 2°	7 (7%)
Limb. valgus 3°	1 (1%)
Limb. valgus 4°	1 (1%)
Limb. varus 1°	26 (26%)
Limb. varus 2°	18 (18%)
Limb. varus 3°	3 (3%)
Limb. varus 4°	1 (1%)

In our study, the use of RA-TKA significantly reduced the need for soft tissue release, particularly when compared to historical data from conventional TKR. The historical data indicates that in conventional procedures, 29% of patients (*P *= 0.0047) with varus deformities and 84% of patients (*P *< 0.0001) with valgus deformities required soft tissue release [10-13]. This reduction in our study can be attributed to the precision and preoperative planning facilitated by robotic systems, which allow for better alignment and balance without extensive soft tissue manipulation (Table 5).

**Table 5:** Comparison of soft-tissue release in varus and valgus knees in patients undergoing robotic-assisted total knee arthroplasty (RA-TKA) with the Cuvis Joint robot (this study) versus historical data from conventional total knee arthroplasty (CTKA) cohorts. CTKA cohorts: [10-13].

Study type	Varus deformity soft-tissue release	*P*-value (Chi-square test)	Valgus deformity soft-tissue release	*P-*values (Chi-square test)
Our study (RA-TKA)	12.77% (*N *= 12/94)	0.0047	16.67% (*N* = 1/6)	<0.0001
Historical data (CTKA) [10-13]	29%		84%	

Clinical implications

**Figure 4:**
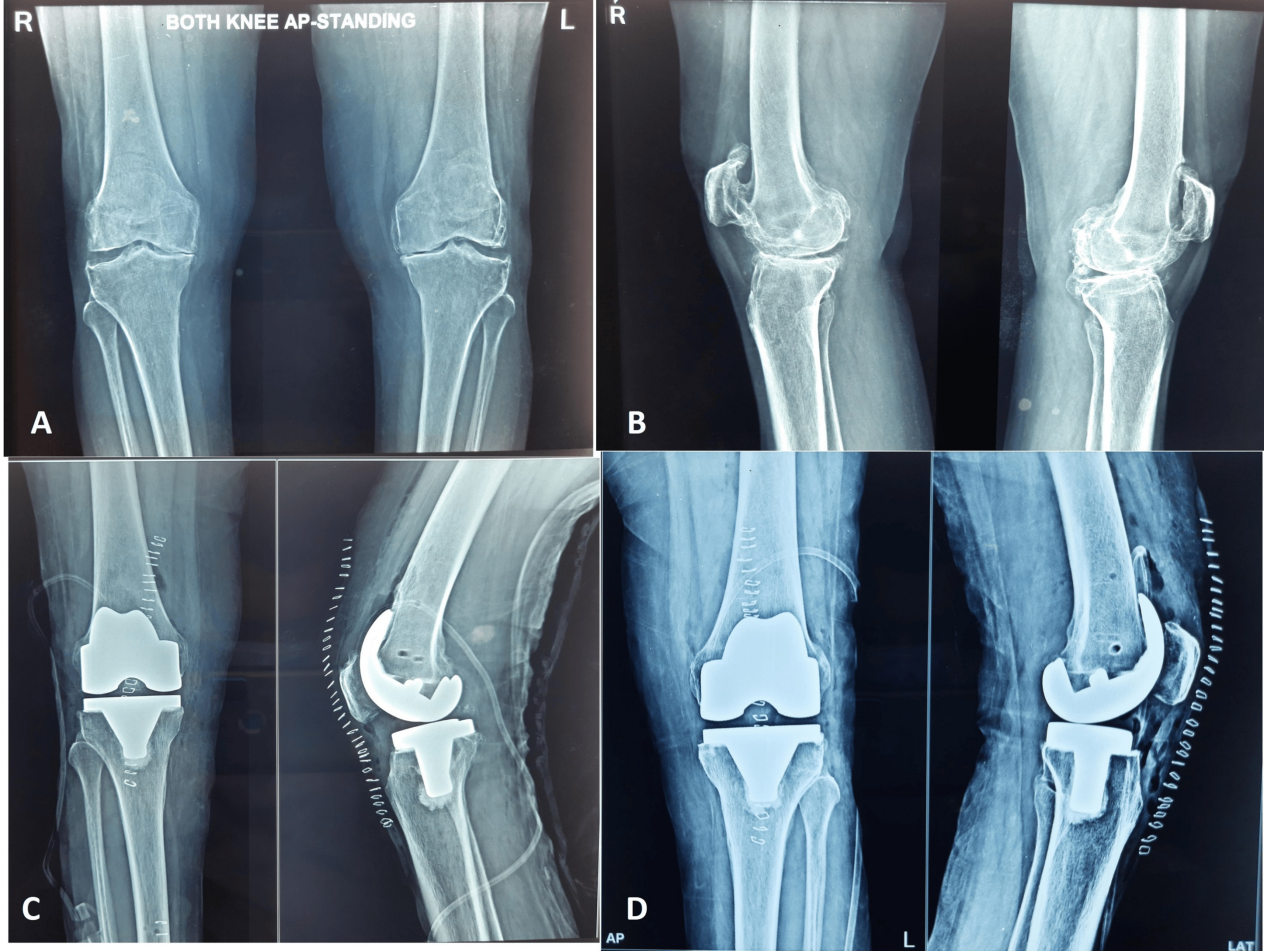
(A and B) Preoperative anteroposterior view of the diseased knee; (C and D) postoperative right and left knees, respectively, implanted using the fully automated Cuvis joint robot system.

Preoperative and postoperative functional and clinical assessments were performed using Knee Society Score (KSS) scale and improvement in ROM after the robotic TKA (Table 6). Poor preoperative clinical and functional KSS (25.76 ± 13.57 and 30.76 ± 10.53, respectively) improved at six weeks and six months follow-up (*P *< 0.001) (Table 6). Mean ROM at six months was reported to be 121.8° ± 5.4°, which was significant when compared to their preoperative values (93.8° ± 15.3°) (*P *< 0.001) (Figure 4).

**Table 6:** Functional and clinical outcomes.

Outcomes (*N* = 100)	Preoperative	Six weeks	Six months	*P*-value (Student’s t-test) (preoperative vs. six months)
Range of motion (degrees)	93.8 ± 15.3	120.4 ± 9.3	121.8 ± 5.4	<0.0001
Knee Society Score (KSS)				
Clinical KSS	25.76 ± 13.57	79.33 ± 7.64	88.12 ± 4.35	<0.0001
Functional KSS	30.76 ± 10.53	88.23 ± 6.25	90.05 ± 2.35	<0.0001

Complication was minimal in our cohort as one patient had superficial infection, which was managed by antibiotics and secondary suturing. No other complications were noted in any patients.

## Discussion

The comprehensive analysis of 100 consecutive patients undergoing TKA with a PS knee design using a fully automated robotic system, guided by preoperative CT scans and planning software, provides valuable insights into the potential benefits of this innovative approach. The results of our study revealed a reduced need for soft tissue release in both varus and valgus knee deformities, highlighting the promising implications for the field of orthopedic surgery.

In accordance with the study by Clark et al. [14] our investigation into TKA with a fully automated robotic system reveals immediate advantages. The noteworthy decrease in soft tissue releases leads to enhanced ROM.

The observed decline in the necessity for soft tissue release, in both varus and valgus knee deformities, aligns seamlessly with the contemporary goals of TKA procedures. Preservation of soft tissue integrity is paramount for improved postoperative function, given the associated complications such as instability and increased risk of revision surgery [4,14]. The requirement for the release of collateral ligaments to achieve a stable, balanced TKA has been reported to arise in about 50%-100 % of procedures [11].

In a prospective cohort analysis of 175 consecutive RA-TKA patients, it was reported that 29.7% of the patients required soft tissue release for varus knees [10]. Among patients with valgus deformity ranging from 6° to 12°, the rate of release needed was 22% and for the patients with valgus deformity of 12° or greater, the rate of release required was 67% [10]. According to another study, 76% of knees (62 out of 82) needed to have medial collateral ligament release to treat varus deformity [12]. Research on 214 cases of valgus knee deformity found that the overall incidence of release in the valgus knee is 100%. The study also reported that 15% of knees required one release, whereas 32% required two releases, 28% required three releases, and 14% required four or more releases [13]. The proportion of soft tissue release in our cohort was significantly lower with pre-operative varus deformity ranging from 7° to 18° and valgus deformity ranging from 6° to 14°. Varus knees (12.77% on comparison with historic control of 29%, p value=0.0047) and 1 out of 6 valgus knees (16.67%, p-value <0.0001 on comparison with historic control of 84%) [10-13] required posteromedial release and posterolateral release respectively to achieve a well-balanced knee.

Our results emphasize the importance of incorporating pre-operative CT scans and planning software into robotic TKA workflows. This integration enhances precision in bone resection and implant placement, reducing the need for extensive soft tissue release to achieve optimal knee balance. A longitudinal data collection series was initiated in a joint arthroplasty registry under the supervision of a single surgeon. The study included 120 patients undergoing RA-TKA, compared with a historical cohort of 100 patients who underwent conventional TKA (CI-TKA). In the RA-TKA group, 7.5% (9 cases) required formal soft tissue release, significantly lower than the 46% (46 cases) observed in the CI-TKA group [15]. Our study aligns with this previous study as a lower incidence of soft tissue releases with RA-TKA was observed in our cohort. These findings underscore the effectiveness of robotic-assisted technology in reducing the incidence of soft tissue releases during knee arthroplasty.

The incorporation of robotic systems, coupled with preoperative planning, introduces a heightened level of precision and individualization in TKA procedures [16]. This personalized approach becomes particularly crucial when addressing the diverse anatomical complexities presented by patients with varying degrees of varus and valgus deformities. The technology empowers surgeons to tailor the surgical plan based on each patient's unique anatomy, leading to improved overall alignment and increased implant longevity.

The finding suggests that the majority of varus deformities were adequately addressed through pre-operative planning and robotic assistance, minimizing the need for additional soft tissue intervention. This further supports the efficacy of the preoperative planning and robotic system in addressing even less common deformities without resorting to extensive soft tissue release.

Our study demonstrates that the utilization of RA-TKA yielded intraoperative compartment balance in extension, while significantly enhancing flexion balancing. In this study, RA-TKA exhibited a greater degree of symmetry in the medial and lateral compartments, as well as a reduced occurrence of excessive stuffing during flexion. In contrast to jig-based TKA, numerous studies have consistently demonstrated that robotic TKA yields superior outcomes in terms of precise implant positioning and dependable restoration of the mechanical axis [17]. While some surgeons believe that short-term improvements in surgical precision do not exhibit a significant correlation with enhanced clinical outcomes or reduced rates of complications. Indeed, certain studies have demonstrated an elevated incidence of complications within the cohort, including pin site fractures, as well as extended durations of operating room procedures [18,19]. However, we did not observe any such complications including pin-site infection, any variations from the preplanned values, or any implant failure during the follow-up. This study demonstrated a significant decrease in the necessity for soft tissue release when compared to historical data from conventional TKA procedures. One reason for the higher rate of soft tissue release in CTKA could be the tendency to perform early collateral ligament releases in all deformed knees during the initial exposure to achieve neutral alignment. However, by utilizing pre-operative planning and robotic-assisted software for TKA in our approach, we found that early ligament release was often unnecessary.

The utilization of RA-TKA, guided by preoperative CT planning, offers promising advancements in achieving optimal knee balance, minimizing soft tissue release, and enhancing precision, thereby contributing to improved postoperative outcomes in the field of orthopedic surgery.

Clinical relevance

The potential clinical consequences of minimizing the necessity for soft tissue release are noteworthy, as it has the potential to expedite postoperative recovery, enhance functional results, and reduce the incidence of problems linked to substantial manipulation of soft tissues [8]. This method is in line with the prevailing trend in orthopedic surgery, which favors minimally invasive approaches that facilitate faster recovery and improve patient satisfaction [5].

The utilization of RA-TKA offers the potential to minimize the need for excessive soft tissue release, hence optimizing the alignment of the knee in the coronal plane concerning the mechanical axis, ultimately leading to a well-balanced knee.

Limitations

This study has a few limitations. First, there is the absence of control cohorts and comparison with historical controls. Second, the cohort of patients with valgus knee deformities is small. Third, the Knee Society Score was assessed over a limited period of 6 months. Despite these constraints, the study employed a reproducible technique to establish a baseline for the required soft tissue release with flexion and extension gap balancing in robot-assisted TKA. These patients are being continuously monitored to evaluate patient-reported outcomes at longer follow-ups.

## Conclusions

In conclusion, the findings of this study underscore the potential of preoperative CT scans and planning software in conjunction with robotic assistance to reduce the need for soft tissue release in TKA. This integrated approach represents a significant step forward in achieving precise and individualized knee arthroplasty, with potential implications for enhanced postoperative outcomes and patient satisfaction.

The reduced need for soft tissue release in both varus and valgus knee deformities underscores the potential clinical benefits of employing preoperative CT scans and planning software in conjunction with RA-TKA. This approach not only enhances the precision of implant placement but also minimizes the disruption of surrounding soft tissues, which is crucial for postoperative function and long-term implant durability.
